# Ultra-Processed Food Intake and Postpartum Depressive Symptoms in Early Postpartum: A Pilot Cross-Sectional Study in Greece

**DOI:** 10.3390/nu18081191

**Published:** 2026-04-10

**Authors:** Aikaterini Mavroudi, George Panayiotou, Thalia Bellali, Maria Kantilafti, Stavri Chrysostomou

**Affiliations:** 1 Department of Life Sciences, School of Sciences, European University Cyprus, 6 Diogenes Str., Engomi 2404, P.O. Box 22006, Nicosia 1516, Cyprus; km241074@students.euc.ac.cy (A.M.); g.panayiotou@euc.ac.cy (G.P.); m.kantilafti@euc.ac.cy (M.K.); 2 Department of Nursing, International Hellenic University, 57001 Thessaloniki, Greece; t.bellali@external.euc.ac.cy

**Keywords:** ultra-processed foods, postpartum depression, edinburgh postnatal depression scale, early postpartum, diet quality, sleep quality, breastfeeding, cross-sectional study

## Abstract

**Background/Objectives:** Emerging evidence suggests that ultra-processed food (UPF) consumption may be associated with depressive symptoms, yet data in the early postpartum period remain limited. This pilot study aimed to examine the association between UPF intake and postpartum depressive symptoms among women in the early postpartum. **Methods:** In this cross-sectional pilot study, 137 women within 6–8 weeks postpartum were recruited from hospitals, maternity clinics, and online support networks in Greece. Dietary intake was assessed using a validated food frequency questionnaire and classified according to the NOVA system. UPF consumption was categorized into quartiles. Postpartum depressive symptoms were evaluated using the Edinburgh Postnatal Depression Scale (EPDS), with clinically significant symptoms defined as EPDS ≥ 13. Multivariable logistic regression analyses were conducted, adjusting for sociodemographic and lifestyle factors. **Results:** The prevalence of clinically significant postpartum depressive symptoms was 29.9%. No statistically significant associations were observed in adjusted models. However, a higher prevalence of depressive symptoms was observed among women in the highest UPF intake quartile (40.0%) compared with lower quartiles (25.7–28.1%). Poor sleep quality was independently associated with higher odds of depressive symptoms, whereas breastfeeding was associated with lower odds. Confidence intervals were wide, indicating limited statistical precision due to the small sample size. **Conclusions:** While no statistically significant association was observed in multivariable analyses, a higher prevalence of depressive symptoms was noted among women in the highest UPF intake quartile. The wide confidence intervals indicate substantial uncertainty, and the findings should be interpreted with caution. Larger, adequately powered studies are required to confirm these findings.

## 1. Introduction

Postpartum depression (PPD) is a serious mental health disorder recognized in the DSM-IV and DSM-5 as a form of major depressive disorder with perinatal onset, with symptoms emerging during pregnancy or within the first 4–6 weeks after delivery and potentially persisting for years [1,2,3]. Globally, the prevalence of PPD is estimated at 17.22% (95% CI: 16.00–18.05), with up to 15% during the first year postpartum across eighty countries and regions [4]. Across Europe, rates vary by region—about 16.34% in Southern and 13.78% in Northern Europe—while in Greece, early postpartum prevalence ranges between 12% and 14.5%, declining in subsequent months [5,6,7,8].

PPD is characterized by persistent low mood, anhedonia, fatigue, sleep and appetite disturbances, guilt, poor concentration, irritability, anxiety, and suicidal ideation, mirroring symptoms of major depressive disorder [9]. The etiology of PPD is multifactorial, involving social, psychological, and biological factors [10]. Social risk factors include limited social support, intimate partner violence, financial strain, young maternal age, low education or income, and migration [11,12,13]. Furthermore, biological contributors include obesity, inflammation, obstetric complications, and genetic or epigenetic variations [10,14]. In addition, psychological factors such as prior mood disorders, antenatal anxiety, and stressful life events further increase susceptibility for PPD [15,16,17].

Emerging evidence suggests a role of modifiable lifestyle factors, particularly diet, in the prevention and management of PPD. Nutrition influences inflammation, oxidative stress, gut microbiota, epigenetic regulation, and neuroplasticity [18]. These biological pathways are directly implicated in the pathophysiology of depressive disorders. Diets with higher inflammatory potential may increase circulating pro-inflammatory cytokines, which can affect neurotransmitter metabolism and hypothalamic–pituitary–adrenal (HPA) axis regulation [10,19]. In addition, alterations in gut microbiota composition may influence mood through gut–brain axis signaling and immune activation [20]. Given the substantial hormonal and immune adaptations occurring during the postpartum period, these diet-related mechanisms may be particularly relevant to postpartum depressive symptomatology [10,17]. Accordingly, healthier dietary patterns, such as high fruit and vegetable intake and reduced consumption of red meat and fast food, are associated with lower depressive symptomatology. Moreover, specific nutrients—including folate, vitamins B6 and B12, omega-3 fatty acids, zinc, selenium, and magnesium—may also be protective [21,22]. However, studies suggest that dietary patterns, rather than individual nutrients, provide a more comprehensive understanding [23]. Notably, a Greek cohort showed that adherence to a “healthy” dietary pattern during pregnancy has been linked with reduced postpartum depressive symptoms [24].

Ultra-processed foods (UPF)—industrial formulations high in added sugars, fats, and salt, and low in fiber and micronutrients—are an emerging dietary concern [25]. According to the NOVA classification, UPF consumption has increased sharply worldwide, contributing to poor diet quality and higher intake of added sugars [26]. Mechanistically, UPF may displace nutrient-dense foods, increase glycemic load, alter gut microbiota, and promote systemic inflammation [27,28]. Observational studies have reported associations between UPF consumption and depressive symptoms, anxiety, and stress [29,30,31]. During pregnancy, UPF consumption has been linked with excessive gestational weight gain and gestational diabetes [32]. Although emerging evidence has examined UPF consumption during pregnancy in relation to depressive symptoms, considerably less attention has been given to dietary exposure during the early postpartum period. The postpartum phase represents a distinct biological and psychosocial context, characterized by hormonal fluctuations, immune adaptations, sleep disruption, and caregiving demands, which may modify the relationship between dietary intake and mood. Accordingly, investigating UPF intake specifically in the early postpartum period is warranted. Therefore, the present cross-sectional pilot study aimed to examine the association between UPF intake during the early postpartum period and postpartum depressive symptoms at 6–8 weeks after delivery, as assessed by the Edinburgh Postnatal Depression Scale (EPDS). We hypothesized that higher UPF intake would be associated with greater odds of clinically significant postpartum depressive symptoms (EPDS ≥ 13), given evidence linking UPF consumption with poorer diet quality as well as inflammatory and metabolic pathways implicated in depression.

## 2. Materials and Methods

### 2.1. Study Design and Setting

This pilot cross-sectional study aimed to assess the feasibility of the research protocol and explore preliminary associations between UPF consumption in early postpartum and postpartum depressive symptoms.

The study was conducted in Thessaloniki, Greece, between July and October 2025. Participants were recruited from private maternity clinics and through postpartum online support groups using a convenience sampling approach. The study followed an observational, non-interventional design, and data were collected using self-administered online questionnaires.

Participation was voluntary, and all participants provided informed electronic consent prior to completing the questionnaire. Participants were informed about the purpose of the study and their right to withdraw at any time without consequences. All responses were collected anonymously, and no identifiable personal data was recorded. Data confidentiality and anonymity were ensured in compliance with the General Data Protection Regulation (EU Regulation 2016/679).

### 2.2. Participants and Sampling

A total of 137 postpartum women participated in this pilot study. Eligible participants were women aged 18 years or older who had given birth within the previous 6–8 weeks, were residents of Thessaloniki, Greece, and were able to read and complete self-administered questionnaires in Greek. Women with a history of major psychiatric disorders (e.g., bipolar disorder or psychosis), pre-existing depression or anxiety disorders diagnosed prior to pregnancy, chronic endocrine or metabolic conditions known to affect mood (e.g., hypothyroidism), or those taking antidepressant medication during pregnancy were excluded.

Participants were recruited via convenience sampling from hospitals, maternity clinics, and online postpartum support networks. Eligibility criteria were assessed using screening questions, and measures were taken to minimize duplicate responses (e.g., response checks). An a priori power calculation for the planned large-scale cross-sectional study indicated that a total sample of 488 participants would be required to detect an assumed odds ratio of 1.7 for clinically significant postpartum depressive symptoms (EPDS ≥ 13) comparing the highest versus the lowest UPF intake, with 80% power at α = 0.05 [33]. The present pilot study included 137 participants to assess feasibility and generate preliminary effect size estimates.

### 2.3. Data Collection

Data were collected using a structured online questionnaire administered via Google Forms. The questionnaire consisted of five sections assessing sociodemographic and obstetric characteristics, dietary intake, depressive symptoms, physical activity, and sleep quality. The estimated completion time was approximately 20–25 min.

### 2.4. Sociodemographic and Obstetric Characteristics

Sociodemographic information included age, educational level, employment status, marital status, family income, smoking habits, and parity. Obstetric data included mode of delivery and the presence of gestational complications, such as gestational diabetes mellitus and hypertensive disorders of pregnancy. Gestational diabetes was classified according to the World Health Organization (WHO) criteria [34], and gestational weight gain was categorized based on the Institute of Medicine (IOM) guidelines [35]. Pre-pregnancy BMI category was self-reported by participants, who selected the category corresponding to their BMI range (underweight, normal weight, overweight, or obese) according to the World Health Organization (WHO) BMI classification.

### 2.5. Depressive Symptoms

Postpartum depressive symptoms were assessed using the Edinburgh Postnatal Depression Scale (EPDS), a 10-item self-report instrument designed to evaluate maternal mood during the previous seven days [36]. Total scores range from 0 to 30, with a cut-off score of ≥13 indicating clinically significant depressive symptoms. The EPDS has been validated for use in the Greek population and demonstrates high internal consistency [37,38]. Because the EPDS includes an item related to self-harm ideation, participants were provided with information regarding available mental health support services and were encouraged to seek professional consultation if needed.

### 2.6. Dietary Intake

Dietary intake during the previous 30 days, corresponding to the early postpartum period (6–8 weeks after delivery), was assessed using a semi-quantitative Food Frequency Questionnaire (FFQ) validated for the Greek population [39]. Participants completed the questionnaire at 6–8 weeks postpartum. The FFQ captured the frequency of consumption of 69 food items across six response categories, ranging from “rarely/never” to “≥2 times per day,” with each item linked to a standard portion size. Responses were coded on an ordinal scale from 0 to 5 (0 = rarely/never, 1 = 1–3 times/month, 2 = 1–2 times/week, 3 = 3–6 times/week, 4 = once/day, 5 = ≥2 times/day), with higher scores indicating higher consumption frequency. These scores were treated as continuous variables for the purposes of analysis.

Food items were classified according to the NOVA food classification system into four groups based on the degree of industrial processing [40]. UPF intake (NOVA group 4) was operationalized using a frequency-based index calculated by summing the reported consumption frequencies of all items classified as NOVA group 4, as estimating UPF intake as a percentage of total energy intake was not feasible given the structure of the FFQ and the pilot nature of the study. Although standard portion sizes (e.g., grams or household measures) were provided for each food item, the FFQ was semi-quantitative and designed to capture consumption frequency using predefined categories rather than precise intake. Therefore, it does not allow accurate estimation of total energy intake or the calculation of UPF consumption as a percentage of total energy. Classification was based on established NOVA guidelines and relevant literature. In cases of ambiguity, items were reviewed and discussed among the research team until consensus was reached. Quartiles were determined from the distribution of the UPF index in the study sample. Inter-rater reliability was not formally assessed and is acknowledged as a limitation. The NOVA classification system has been widely applied in epidemiological studies examining associations between UPF consumption and various health outcomes [25,30].

### 2.7. Physical Activity

Physical activity was assessed using the International Physical Activity Questionnaire–Short Form (IPAQ-SF), which has been validated for use in the Greek population [41]. The questionnaire estimates weekly energy expenditure expressed as metabolic equivalent task minutes per week (MET-min/week). Participants were categorized as having low (≤600 MET-min/week), moderate (600–3000 MET-min/week), or high (>3000 MET-min/week) levels of physical activity according to established IPAQ scoring guidelines.

### 2.8. Sleep Quality

Sleep quality was evaluated using the Pittsburgh Sleep Quality Index (PSQI) [42]. The PSQI assesses seven components of sleep behavior, including duration, latency, efficiency, and disturbances, yielding a global score ranging from 0 to 21, with scores > 5 indicating poor sleep quality. The Greek version of the PSQI has demonstrated excellent reliability and validity [43].

### 2.9. Statistical Analysis

All statistical analyses were performed using IBM SPSS Statistics version 29.0 (IBM Corp., Armonk, NY, USA). Continuous variables were summarized as mean ± standard deviation (SD) for normally distributed data or median (interquartile range) for non-normally distributed data, while categorical variables were presented as frequencies and percentages. Prior to statistical analyses, the dataset was manually screened for data entry errors, missing values, and inconsistencies in response format. Minor data cleaning procedures included standardizing response formats (e.g., converting gestational age responses to a common unit of weeks where needed), without modifying or imputing the underlying values. Normality was assessed using the Kolmogorov–Smirnov test.

For analytical purposes, participants were categorized according to postpartum depressive symptom status based on the Edinburgh Postnatal Depression Scale (EPDS) cut-off score (EPDS ≥ 13 vs. <13). Comparisons between groups were conducted using independent-samples *t*-tests or Mann–Whitney U tests for continuous variables, as appropriate, and χ^2^ tests for categorical variables.

Binary logistic regression was used to examine the association between postpartum UPF intake (frequency-based NOVA group 4 index, categorized into quartiles) and clinically significant postpartum depressive symptoms (EPDS ≥ 13) at 6–8 weeks postpartum. The lowest quartile of UPF intake served as the reference category. Crude and adjusted odds ratios (ORs) with 95% confidence intervals (CIs) were estimated. Multivariable models were a priori adjusted for maternal age, body mass index (BMI) category, educational level, parity, physical activity, and sleep quality, based on their established relevance as potential confounders in the association between diet and postpartum depressive symptoms. These covariates were selected a priori based on existing literature; however, some relevant psychosocial variables, such as social support and detailed mental health history, were not measured. Multicollinearity was assessed using variance inflation factors (VIFs). In addition, breastfeeding status was examined in a sensitivity analysis, given prior evidence supporting its association with postpartum depressive symptoms [44] and its potential relevance to maternal behaviors in the early postpartum period. This additional model was conducted to assess whether adjustment for breastfeeding materially altered the observed associations between UPF intake and depressive symptoms. Missing data were minimal (<1%), and complete-case (listwise) analysis was performed for multivariable models. Model fit was assessed using pseudo-R^2^ indices (e.g., Nagelkerke R^2^). Statistical significance was set at *p* < 0.05.

## 3. Results

### 3.1. Participant Characteristics

A total of 137 postpartum women participated in the study. The mean age of participants was 32.5 years. The majority held a tertiary education degree (83.2%), were married (97.1%), and were employed full-time (73.7%). In terms of household income, approximately half of them (46.7%) reported an annual income between €10,000–20,000. Also, more than half of the participants (52.6%) reported never having smoked, while the rest were former smokers and current smokers.

Regarding obstetric and perinatal characteristics, slightly more than half of the participants were primiparous (54.0%), while 46.0% had one or more previous pregnancies. Cesarean section was reported by 48.9%. With respect to maternal health characteristics, a small percentage (16.1%) of women were diagnosed with gestational diabetes mellitus. Pre-pregnancy BMI was normal in 69.3% of participants, while 19.7% were overweight and 5.8% were obese (Table 1). Regarding gestational weight gain (GWG), almost two-thirds of participants (72.3%) had adequate gestational weight gain, while the remaining 27.8% had excessive or insufficient weight gain. Most women were currently breastfeeding (83.9%), and a very small percentage of them (6.6%) had experienced a preterm birth. These findings are presented in Table 1.

Regarding health-related variables, nearly half of the participants reported moderate physical activity levels according to the IPAQ (48.2%), and only 12.4% reported high physical activity levels. Poor sleep quality, as assessed by the PSQI, was reported by 76.6% of the sample. Participants were approximately evenly distributed across UPF intake quartiles, with about one quarter of the sample in each quartile. Overall, 29.9% of women screened positive for postpartum depressive symptoms (EPDS ≥ 13). All these results are shown in Table 2.

### 3.2. UPF Consumption Across Quartiles

Analysis of ultra-processed food consumption by intake quartile revealed differences in consumption patterns (Table 3), with a more detailed breakdown of individual ultra-processed food items in Table A1. Frequently consumed UPF items included mayonnaise-based salads, full-fat chocolate, rusks and white bread, and processed meat products. As expected, participants in higher quartiles (Q3 and Q4) reported markedly higher consumption frequencies of nearly all UPF categories. Further examination of individual UPF items revealed statistically significant differences in consumption patterns across quartiles. Items such as white bread, breakfast cereals, low-fat processed meats, ready-to-eat savory pies, pizza, croissants, chocolate, and ice cream showed consistent increases in mean frequency from Q1 to Q4, with statistically significant differences across quartiles (Table 3).

### 3.3. Associations Between UPF Intake and Depressive Symptoms

**Figure 1:**
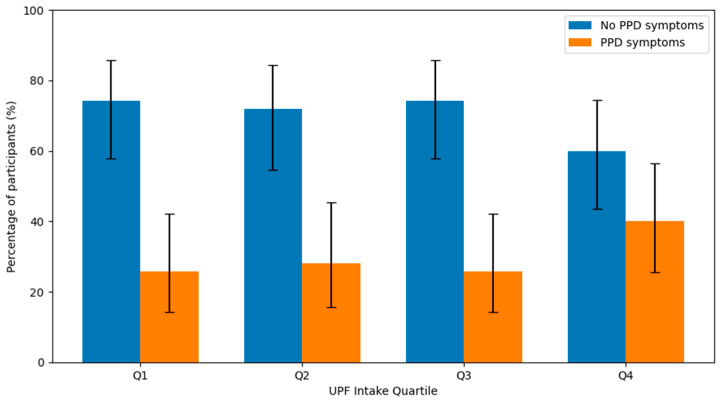
Distribution of Participants with and without Postpartum Depressive Symptoms (EPDS ≥ 13) Across UPF Intake Quartiles. Error bars represent 95% binomial confidence intervals.

Figure 1 illustrates the distribution of participants with and without postpartum depressive symptoms across UPF intake quartiles. The proportion of women screening positive for postpartum depressive symptoms (EPDS ≥ 13) ranged from 25.7% in Q1 to 40.0% in Q4. Specifically, 9 out of 35 women (25.7%) in Q1, 9 out of 32 (28.1%) in Q2, 9 out of 35 (25.7%) in Q3, and 14 out of 35 (40.0%) in Q4 screened positive for postpartum depressive symptoms. A higher prevalence was observed in the highest UPF intake quartile; however, this difference did not reach statistical significance (*p* = 0.506).

Crude associations between UPF consumption and depressive symptoms were examined using logistic regression without adjustment for covariates. Using the lowest quartile (Q1) as the reference, no statistically significant associations were observed across higher quartiles. The odds of scoring above the EPDS threshold (≥13) were not significantly different in Q2 (OR = 1.13, 95% CI: 0.38–3.33, *p* = 0.824), Q3 (OR = 1.00, 95% CI: 0.34–2.92, *p* = 1.000), or Q4 (OR = 1.93, 95% CI: 0.70–5.32, *p* = 0.206). In Model 1, adjusted for maternal age, BMI category, educational level, sleep quality, physical activity, and parity, UPF intake was not significantly associated with clinically significant postpartum depressive symptoms (overall *p* = 0.644). Compared with the lowest UPF intake quartile, the odds ratio for EPDS ≥ 13 was 1.77 (95% CI: 0.58–5.43) in the highest quartile. The point estimate was above 1; however, the confidence interval was wide and included the null value, indicating substantial uncertainty. Poor sleep quality was independently associated with higher odds of postpartum depressive symptoms (OR = 5.17, 95% CI: 1.42–18.82, *p* = 0.013). No statistically significant associations were observed for maternal age, educational level, BMI category, physical activity, or parity (Table 4).

In Model 2, additionally adjusting for breastfeeding status, UPF intake remained non-significantly associated with depressive symptoms (Q4 vs. Q1: OR = 1.45, 95% CI: 0.44–4.80, *p* = 0.548). Breastfeeding itself was independently associated with lower odds of postpartum depressive symptoms (OR = 0.16, 95% CI: 0.05–0.51, *p* = 0.002). The association between poor sleep quality and depressive symptoms remained robust after this additional adjustment (OR = 6.66, 95% CI: 1.63–27.21, *p* = 0.008) (Table 4). Logistic regression model fit was acceptable (Hosmer–Lemeshow *p* = 0.838), with Nagelkerke R^2^ = 0.269. The model correctly classified 75.2% of cases (vs. 69.9% in the null model). When UPF quartiles were modeled as an ordinal variable in the multivariable-adjusted logistic regression model (Model 1), no statistically significant trend was observed across quartiles (*p* for trend = 0.383).

## 4. Discussion

The present pilot cross-sectional study examined the association between UPF intake in the early postpartum period and postpartum depressive symptoms. A higher prevalence of postpartum depressive symptoms was observed among women in the highest UPF intake quartile (40.0%) compared with those in the lower quartiles (25.7–28.1%); however, this difference was not statistically significant. Confidence intervals were wide, reflecting the limited number of outcome events in this pilot sample and indicating substantial uncertainty. No evidence of substantial multicollinearity was observed in adjusted models. Moreover, poor sleep quality was independently associated with higher odds of postpartum depressive symptoms, whereas breastfeeding was associated with significantly lower odds of postpartum depressive symptoms. The characteristics of the study sample should be considered when interpreting these findings, particularly regarding potential selection bias and limited generalizability. Participants were recruited through private clinics and online postpartum support groups using a convenience sampling approach, which may have resulted in a sample with higher educational attainment and limited representativeness. This may introduce selection bias and limit the generalisability of the findings to the broader Greek postpartum population.

Growing evidence in perinatal populations suggests that maternal dietary patterns may influence the risk of postpartum depressive symptoms. In a large cross-sectional study of Iranian women (*n* = 1028), high adherence to a western dietary pattern was associated with a significantly increased likelihood of high postpartum depressive symptoms (OR = 2.67), whereas adherence to a prudent dietary pattern was inversely associated (OR = 0.55) [45]. Similarly, among Chinese lactating women, poorer overall dietary quality assessed using the Diet Balance Index was positively associated with postpartum depressive symptoms [46]. Although these studies primarily evaluated dietary patterns or overall diet quality rather than UPF consumption per se, they collectively suggest that lower-quality, nutritionally imbalanced diets during the perinatal period may contribute to depressive symptomatology. In parallel, emerging data from pregnant populations indicate that higher UPF intake is associated with greater prevalence of depressive symptoms [47], supporting the hypothesis that food processing level may be an additional relevant dimension in maternal mental health.

Consistent with this pattern, the odds ratio in the highest quartile was greater than 1; however, the wide confidence interval indicates substantial uncertainty. As shown in Table 3, women in the highest UPF intake quartile reported higher consumption of refined grain products, processed meats, and sweet bakery items. These food groups are characteristic components of Western-type dietary patterns, which have previously been associated with higher depressive symptomatology [31,48]. Such dietary profiles are generally lower in micronutrient density and higher in refined carbohydrates and saturated fats, factors that may increase inflammatory potential [19]. These findings are broadly consistent with prior evidence linking higher UPF intake with depressive symptoms [29,30,31]. Taken together, these results provide preliminary estimates in an exploratory context within an understudied area and underscore the need for adequately powered longitudinal research to clarify the temporal nature of the association between UPF intake and postpartum depressive symptoms.

Beyond dietary factors, poor sleep quality was significantly associated with increased odds of postpartum depressive symptoms in the adjusted model. This finding is consistent with prior evidence demonstrating a robust relationship between sleep disturbances and perinatal depression, including the postpartum period [49]. Prospective studies have further shown that poor sleep quality in early postpartum independently predicts subsequent depressive symptoms [50], while cross-sectional research has reported substantially higher odds of postpartum depression among women with poor sleep quality [51]. These findings reinforce the established role of sleep disruption as an important modifiable factor in postpartum mental health [49,50,51].

With regard to breastfeeding, our findings indicate that although breastfeeding was independently associated with lower odds of postpartum depressive symptoms in the sensitivity model (OR = 0.16, 95% CI: 0.05–0.51, *p* = 0.002), its inclusion did not materially change the magnitude or direction of the association between UPF intake and depressive symptoms. This suggests that breastfeeding status did not confound the association between UPF intake and postpartum depressive symptoms in this study. The observed protective association is consistent with previous studies reporting lower odds of postpartum depressive symptoms among breastfeeding women, although the relationship has been described as complex and potentially bidirectional [52,53,54,55], and reverse causation cannot be excluded, as depressive symptoms may influence the initiation or continuation of breastfeeding.

Several biological mechanisms may plausibly explain the observed associations among dietary quality, food-processing level, and depressive symptomatology during the perinatal period. Prospective evidence indicates that dietary patterns with higher inflammatory potential are associated with increased risk of incident depression [19]. Conversely, greater adherence to Mediterranean-style dietary patterns has been consistently associated with reduced risk of depressive outcomes in both observational and interventional studies [48,56]. These protective effects are thought to be mediated through anti-inflammatory and antioxidant pathways. In addition, mechanistic research highlights the role of the gut–brain axis in the pathophysiology of depression, suggesting that dietary components may influence mood through alterations in gut microbiota composition, immune activation, and neuroinflammatory signaling [20]. Such mechanisms may be particularly relevant in the postpartum period, which is characterized by substantial endocrine shifts, immune adaptations, and increased vulnerability to affective disturbances. Collectively, this biological framework supports the hypothesis that higher intake of UPF—typically characterized by lower nutrient density and greater inflammatory potential—may contribute to depressive symptoms in susceptible populations.

Overall, while the associations observed in this pilot study did not reach statistical significance, emerging evidence from perinatal and general population studies [29,30,31] suggest that UPF consumption warrants further investigation as a potentially modifiable factor in postpartum mental health. Given the substantial burden of postpartum depression and the modifiable nature of dietary exposures, clarifying this relationship in adequately powered longitudinal studies remains a research priority.

### Strengths and Limitations

Nevertheless, this pilot study has notable strengths. Although previous Greek studies have examined overall dietary patterns during pregnancy in relation to postpartum depression [24], to our knowledge, this is the first study in a Greek postpartum population to specifically evaluate UPF intake during the postpartum period using the NOVA classification system. The study utilized validated instruments for both dietary assessment and depressive symptom screening. Importantly, as an exploratory analysis embedded within a larger research project, the present findings provide essential feasibility data and preliminary effect size estimates to inform the design of a future adequately powered prospective investigation.

The present findings should be interpreted in light of several limitations. Given the cross-sectional design, reverse causation cannot be excluded. It is possible that women experiencing depressive symptoms may modify their dietary behaviors, including increased consumption of ultra-processed foods, due to factors such as fatigue, emotional eating, or limited time availability in the early postpartum period. First, the cross-sectional design precludes conclusions regarding temporality or causality. Furthermore, postpartum depressive symptoms were assessed using a screening instrument rather than a clinical diagnostic interview, which may have resulted in misclassification of cases. Second, dietary intake was assessed using a self-reported food frequency questionnaire, which may be subject to recall bias and misclassification, although a validated instrument for the Greek population was employed. Moreover, UPF intake was operationalized using a frequency-based index derived from FFQ data and was not expressed as a percentage of total energy intake. This approach may limit direct comparability with studies that use energy-adjusted or energy-based UPF metrics. In this context, UPF intake was examined at the level of individual food items. Table A1 provides a detailed breakdown of these components, and Table 3 presents selected items; key findings are summarized in the text. Additionally, it may introduce some degree of exposure misclassification compared with energy-based assessments. Third, despite adjustment for key sociodemographic and lifestyle factors, residual confounding cannot be excluded. The relatively small sample size and limited number of cases resulted in wide confidence intervals, reducing statistical precision. Importantly, given the pilot nature of the study (*n* = 137), the analysis was underpowered to detect modest associations (e.g., ORs in the range of approximately 1.5–2.0). Therefore, the absence of statistically significant findings should be interpreted with caution and should not be considered evidence of no association or indicate a directional trend. Additionally, sleep quality and breastfeeding may lie on the causal pathway between postpartum distress, lifestyle constraints, and dietary behaviors; therefore, adjustment for these variables may attenuate or distort the observed associations. Accordingly, the models should be interpreted as exploratory rather than causal.

In addition, participants were recruited through private clinics and online postpartum support groups using a convenience sampling approach. Although recruitment was not restricted to a specific socioeconomic group, the high proportion of tertiary-educated and married participants suggests a relatively socio-demographically advantaged sample. Therefore, the findings may not be fully generalizable to the broader postpartum population. This recruitment approach may preferentially include women with higher health literacy, greater access to healthcare services, and higher digital engagement, thereby underrepresenting more socioeconomically diverse populations.

## 5. Conclusions

In conclusion, this pilot cross-sectional study did not identify statistically significant associations between UPF intake and postpartum depressive symptoms after multivariable adjustment. However, a higher prevalence of depressive symptoms was consistently observed among women in the highest quartile of UPF consumption, although this difference didn’t reach statistical significance. Poor sleep quality emerged as an important correlate of postpartum depressive symptoms, highlighting the multifactorial nature of maternal mental health in the early postpartum period. These preliminary findings, together with emerging evidence linking dietary quality and food-processing levels to mental health outcomes, support the need for further investigation in perinatal populations. Given the modifiable nature of dietary exposures and the substantial public health burden of postpartum depression, adequately powered prospective studies are warranted to clarify temporal relationships and potential mechanisms.

## Figures and Tables

**Table 1:** Sociodemographic, lifestyle, and obstetric characteristics of the study population (N = 137).

Variable	Category	N (%) or Mean ± SD
Age (years)		32.5 ± 4.3
Education	Primary	1 (0.7%)
	Secondary	22 (16.1%)
	Tertiary	114 (83.2%)
Marital status	Married	133 (97.1%)
	In a relationship	4 (2.9%)
Employment	Full-time	101 (73.7%)
	Part-time	8 (5.8%)
	Unemployed	18 (13.1%)
	Homemaker	10 (7.3%)
Household Income (€)	<10,000	24 (17.5%)
	10,000–20,000	64 (46.7%)
	>20,000	49 (35.8%)
Smoking status	Smoker	20 (14.6%)
	Former	45 (32.8%)
	Never	72 (52.6%)
Parity (number of previous pregnancies)	0	74 (54.0%)
	1	42 (30.7%)
	2	13 (9.5%)
	3	6 (4.4%)
	4	1 (0.7%)
	7	1 (0.7%)
Mode of delivery	Cesarean section	67 (48.9%)
	Vaginal	70 (51.1%)
Gestational diabetes diagnosis	Yes	22 (16.1%)
	No	115 (83.9%)
Pre-pregnancy BMI category	Underweight	7 (5.1%)
	Normal	95 (69.3%)
	Overweight	27 (19.7%)
	Obese	8 (5.8%)
Gestational weight gain category	Below recommended	6 (4.4%)
	Normal	99 (72.3%)
	Excessive	32 (23.4%)
Currently breastfeeding	Yes	115 (83.9%)
	No	22 (16.1%)
Preterm birth (<37 weeks)	Yes	9 (6.6%)
	No	128 (93.4%)

BMI: body mass index. Age is presented as mean ± standard deviation (SD); all other variables are presented as *n* (%).

**Table 2:** Health-Related Variables (N = 137).

Variable	Category	N (%)
Physical Activity (IPAQ)	Low	54 (39.4%)
	Moderate	66 (48.2%)
	High	17 (12.4%)
Sleep Quality (PSQI)	Good	31 (22.6%)
	Poor	105 (76.6%)
Ultra-Processed Food Intake (UPF quartiles)	Q1 (lowest)	35 (25.5%)
	Q2	32 (23.4%)
	Q3	35 (25.5%)
	Q4 (highest)	35 (25.5%)
EPDS ≥ 13	No	96 (70.1%)
	Yes	41 (29.9%)

IPAQ: International Physical Activity Questionnaire; PSQI: Pittsburgh Sleep Quality Index; UPF: ultra-processed foods; EPDS: Edinburgh Postnatal Depression Scale. Values are presented as *n* (%). EPDS ≥ 13 indicates clinically significant postpartum depressive symptoms. PSQI data were available for 136 participants.

**Table 3:** Mean consumption frequencies of selected ultra-processed food items across UPF intake quartiles.

Food Item	Quartile 1	Quartile 2	Quartile 3	Quartile 4	*p*-Value
White bread/rusks	1.29 ± 1.53	2.12 ± 1.54	2.83 ± 1.40	3.29 ± 1.34	<0.001
Breakfast cereals, cereal bars	1.17 ± 1.56	1.88 ± 1.26	2.49 ± 1.15	2.80 ± 1.43	<0.001
Sausages, hot dogs	0.17 ± 0.38	0.16 ± 0.45	0.34 ± 0.68	0.66 ± 0.91	0.005
Low-fat processed meats	0.29 ± 0.62	0.94 ± 1.11	0.86 ± 1.12	1.80 ± 1.37	<0.001
Ready-to-eat savory pies	0.29 ± 0.52	0.62 ± 0.61	1.26 ± 0.95	1.54 ± 1.07	<0.001
Pizza	0.83 ± 0.62	0.88 ± 0.66	1.34 ± 0.84	1.74 ± 1.01	<0.001
Croissants, wafers, cookies	0.69 ± 0.68	0.97 ± 0.90	1.63 ± 1.26	2.37 ± 1.06	<0.001
Chocolate (all types)	1.37 ± 1.26	1.69 ± 1.23	2.03 ± 1.10	2.31 ± 1.49	0.016
Ice cream, milkshake, creamy desserts	1.11 ± 1.13	1.53 ± 1.24	1.97 ± 0.92	2.29 ± 1.25	<0.001
Chips, salty snacks	0.57 ± 0.95	0.97 ± 0.93	0.89 ± 0.93	1.63 ± 0.94	<0.001
Fruit juice (packaged)	1.20 ± 1.23	1.59 ± 1.43	1.94 ± 1.19	2.71 ± 1.51	<0.001
Soda/soft drinks	0.11 ± 0.40	0.50 ± 1.08	0.74 ± 1.07	1.49 ± 1.58	<0.001

Values are presented as mean ± standard deviation (SD) of consumption frequency scores. *p*-values were derived from one-way ANOVA comparing mean consumption frequencies across UPF quartiles. All listed items correspond to NOVA Group 4 (ultra-processed foods) and were included in the UPF frequency index. A detailed breakdown of all ultra-processed food items is provided in Table A1.

**Table 4:** Multivariable logistic regression analysis of factors associated with postpartum depressive symptoms (EPDS ≥ 13).

Variable	Model 1 OR (95% CI)	*p*-Value	Model 2 OR (95% CI)	*p*-Value
UPF Quartiles Q1 (Reference) Q2 Q3 Q4				
	-	-	-	-
	1.06 (0.32–3.48)	0.928	1.05 (0.30–3.67)	0.944
	0.91 (0.29–2.86)	0.877	0.98 (0.29–3.28)	0.976
	1.77 (0.58–5.43)	0.318	1.45 (0.44–4.80)	0.548
Age (per year)	0.94 (0.85–1.04)	0.246	0.92 (0.83–1.03)	0.155
Education (≤Secondary vs. Tertiary)	1.02 (0.35–3.02)	0.969	1.11 (0.36–3.42)	0.855
Poor Sleep Quality (vs. Good)	5.17 (1.42–18.82)	0.013	6.66 (1.63–27.21)	0.008
Physical Activity				
High (Reference)	-	-	-	-
Moderate	2.97 (0.57–15.65)	0.199	3.24 (0.57–18.40)	0.184
Low	3.94 (0.73–21.37)	0.112	3.54 (0.60–20.86)	0.162
BMI				
Normal (Reference)	-	-	-	-
Underweight	0.97 (0.16–5.80)	0.976	0.98 (0.15–6.51)	0.985
Overweight	1.57 (0.59–4.17)	0.368	1.37 (0.49–3.79)	0.546
Obese	1.70 (0.29–9.99)	0.555	1.98 (0.32–12.44)	0.465
Primiparous vs. Multiparous	0.85 (0.37–1.94)	0.694	0.89 (0.37–2.13)	0.791
Breastfeeding (Yes vs. No)				
No (Reference)	-	-	-	-
Yes	-	-	0.16 (0.05–0.51)	0.002

Odds ratios (ORs) and 95% confidence intervals (CIs) were estimated using multivariable logistic regression. Model 1 was adjusted for age, education, BMI category, parity, sleep quality, and physical activity; Model 2 was additionally adjusted for breastfeeding status. Q1 served as the reference category for UPF quartiles, and normal BMI was used as the reference category for BMI. Reference categories are indicated in the first row of each variable. All models are based on complete-case (listwise) analysis. Model fit indices: Nagelkerke R^2^ = 0.269; Hosmer–Lemeshow *p* = 0.838; overall classification accuracy = 75.2% (cut-off = 0.5).

## Data Availability

The data presented in this study are available on reasonable request from the corresponding author. The data are not publicly available due to privacy and ethical restrictions.

## Appendix A

**Table A1:** Mean consumption frequencies of all ultra-processed food items across UPF intake quartiles.

Food Item	Quartile 1 (*n* = 35)	Quartile 2 (*n* = 32)	Quartile 3 (*n* = 35)	Quartile 4 (*n* = 35)	*p*-Value
White bread/rusks	1.29 ± 1.53	2.12 ± 1.54	2.83 ± 1.40	3.29 ± 1.34	<0.001
Bread rolls, pies (incl. Thessaloniki bun)	0.86 ± 0.77	1.56 ± 0.98	1.43 ± 0.88	1.97 ± 0.98	<0.001
Crackers, breadsticks	0.77 ± 0.97	1.16 ± 1.25	1.51 ± 1.27	1.89 ± 1.39	0.002
Breakfast cereals, cereal bars	1.17 ± 1.56	1.88 ± 1.26	2.49 ± 1.15	2.80 ± 1.43	<0.001
Cold cuts/deli meats	0.11 ± 0.32	0.09 ± 0.30	0.06 ± 0.24	0.46 ± 1.04	0.016
Sausages, hot dogs	0.17 ± 0.38	0.16 ± 0.45	0.34 ± 0.68	0.66 ± 0.91	0.005
Low-fat processed meats	0.29 ± 0.62	0.94 ± 1.11	0.86 ± 1.12	1.80 ± 1.37	<0.001
Ready-to-eat savory pies	0.29 ± 0.52	0.62 ± 0.61	1.26 ± 0.95	1.54 ± 1.07	<0.001
Pizza	0.83 ± 0.62	0.88 ± 0.66	1.34 ± 0.84	1.74 ± 1.01	<0.001
Croissants, wafers, cookies	0.69 ± 0.68	0.97 ± 0.90	1.63 ± 1.26	2.37 ± 1.06	<0.001
Syrupy sweets (e.g., baklava)	0.11 ± 0.32	0.44 ± 0.67	0.49 ± 0.78	0.74 ± 0.85	0.003
Pastries, tarts	0.31 ± 0.53	0.41 ± 0.67	0.69 ± 0.72	0.94 ± 1.30	0.012
Chocolate (all types)	1.37 ± 1.26	1.69 ± 1.23	2.03 ± 1.10	2.31 ± 1.49	0.016
Ice cream, milkshake, creamy desserts	1.11 ± 1.13	1.53 ± 1.24	1.97 ± 0.92	2.29 ± 1.25	<0.001
Chips, salty snacks	0.57 ± 0.95	0.97 ± 0.93	0.89 ± 0.93	1.63 ± 0.94	<0.001
Fruit juice (packaged)	1.20 ± 1.23	1.59 ± 1.43	1.94 ± 1.19	2.71 ± 1.51	<0.001
Soda/soft drinks	0.11 ± 0.40	0.50 ± 1.08	0.74 ± 1.07	1.49 ± 1.58	<0.001
Light/diet soft drinks	0.20 ± 0.58	1.00 ± 1.41	1.06 ± 1.28	1.26 ± 1.65	0.005
Mayonnaise	0.34 ± 0.73	0.66 ± 0.90	0.80 ± 0.93	1.49 ± 1.27	<0.001
Light mayonnaise	0.20 ± 0.47	0.41 ± 0.91	0.43 ± 0.61	1.17 ± 1.22	<0.001
Margarine	0.14 ± 0.49	0.47 ± 0.84	0.63 ± 1.00	1.17 ± 1.44	<0.001
UPF frequency index (sum of NOVA-4 item scores)	12.14 ± 3.77	20.03 ± 1.53	25.40 ± 1.74	35.71 ± 5.24	-

Values are presented as mean ± standard deviation (SD). *p*-values were derived from one-way ANOVA comparing mean consumption frequency scores across UPF intake quartiles and are presented as descriptive (unadjusted) values and should be interpreted with caution due to multiple comparisons. All listed items correspond to NOVA Group 4 (ultra-processed foods) and were included in the UPF frequency index. Consumption frequencies represent mean scores of ordinal FFQ categories (coded from 0 to 5 as follows: 0 = never/rarely, 1 = 1–3 times/month, 2 = 1–2 times/week, 3 = 3–6 times/week, 4 = once/day, 5 = ≥2 times/day), with higher values indicating higher consumption frequency. The UPF frequency index was calculated as the sum of all NOVA-4 item scores. Similar conclusions were obtained using non-parametric tests (Kruskal–Wallis).

## References

[1] American Psychiatric Association (1994). Diagnostic and Statistical Manual of Mental Disorders.

[2] Crotty F., Sheehan J. (2004). Prevalence and detection of postnatal depression in an Irish community sample. Ir. J. Psychol. Med..

[3] Serati M., Redaelli M., Buoli M., Altamura A.C. (2016). Perinatal Major Depression Biomarkers: A Systematic Review. J. Affect. Disord..

[4] Liu X., Wang S., Wang G. (2022). Prevalence and Risk Factors of Postpartum Depression in Women: A Systematic Review and Meta-analysis. J. Clin. Nurs..

[5] Giakoumaki O., Vasilaki K., Lili L., Skouroliakou M., Liosis G. (2009). The role of maternal anxiety in the early postpartum period: Screening for anxiety and depressive symptomatology in Greece. J. Psychosom. Obstet. Gynaecol..

[6] Gonidakis F., Rabavilas A.D., Varsou E., Kreatsas G., Christodoulou G.N. (2008). A 6-month study of postpartum depression and related factors in Athens Greece. Compr. Psychiatry.

[7] Koutra K., Roumeliotaki T., Kyriklaki A., Kampouri M., Sarri K., Vassilaki M., Bitsios P., Kogevinas M., Chatzi L. (2017). Maternal depression and personality traits in association with child neuropsychological and behavioral development in preschool years: Mother-child cohort (Rhea Study) in Crete, Greece. J. Affect. Disord..

[8] Micha G., Hyphantis T., Staikou C., Valsamidis D., Arnaoutoglou E., Tzimas P., Vlahos N., Daponte A., Grypiotis I., Pappa P. (2022). Prevalence of postpartum depression and antenatal anxiety symptoms during COVID-19 pandemic: An observational prospective cohort study in Greece. Eur. J. Midwifery.

[9] Pearlstein T., Howard M., Salisbury A., Zlotnick C. (2009). Postpartum depression. Am. J. Obstet. Gynecol..

[10] Payne J.L., Maguire J. (2019). Pathophysiological mechanisms implicated in postpartum depression. Front. Neuroendocr..

[11] Bottino M.N., Nadanovsky P., Moraes C.L., Reichenheim M.E., Lobato G. (2012). Reappraising the relationship between maternal age and postpartum depression according to the evolutionary theory: Empirical evidence from a survey in primary health services. J. Affect. Disord..

[12] Öztora S., Arslan A., Çaylan A., Dağdeviren H. (2019). Postpartum depression and affecting factors in primary care. Niger. J. Clin. Pr..

[13] Zhao X., Zhang Z. (2020). Risk factors for postpartum depression: An evidence-based systematic review of systematic reviews and meta-analyses. Asian J. Psychiatry.

[14] Schiller C.E., Meltzer-Brody S., Rubinow D.R. (2015). The role of reproductive hormones in postpartum depression. CNS Spectr..

[15] Agrawal I., Mehendale A.M., Malhotra R. (2022). Risk Factors of Postpartum Depression. Cureus.

[16] Beck C.T. (2001). Predictors of postpartum depression: An update. Nurs. Res..

[17] Yim I.S., Stapleton L.R.T., Guardino C.M., Hahn-Holbrook J., Schetter C.D. (2015). Biological and psychosocial predictors of postpartum depression: Systematic review and call for integration. Annu. Rev. Clin. Psychol..

[18] Marx W., Moseley G., Berk M., Jacka F. (2017). Nutritional psychiatry: The present state of the evidence. Proc. Nutr. Soc..

[19] Sánchez-Villegas A., Ruíz-Canela M., De La Fuente-Arrillaga C., Gea A., Shivappa N., Hébert J.R., Martínez-González M.A. (2015). Dietary inflammatory index, cardiometabolic conditions and depression in the Seguimiento Universidad de Navarra cohort study. Br. J. Nutr..

[20] Foster J.A., McVey Neufeld K.-A. (2013). Gut–brain axis: How the microbiome influences anxiety and depression. Trends Neurosci..

[21] Chong M.F., Wong J.X., Colega M., Chen L.-W., van Dam R.M., Tan C.S., Lim A.L., Cai S., Broekman B.F., Lee Y.S. (2014). Relationships of maternal folate and vitamin B12 status during pregnancy with perinatal depression: The GUSTO study. J. Psychiatr. Res..

[22] Judge M.P., Beck C.T. (2008). Postpartum Depression and the Role of Nutritional Factors. Handbook of Nutrition and Pregnancy.

[23] Hu F.B. (2002). Dietary pattern analysis: A new direction in nutritional epidemiology. Curr. Opin. Lipidol..

[24] Chatzi L., Melaki V., Sarri K., Apostolaki I., Roumeliotaki T., Georgiou V., Vassilaki M., Koutis A., Bitsios P., Kogevinas M. (2011). Dietary patterns during pregnancy and the risk of postpartum depression: The mother–child ‘Rhea’ cohort in Crete, Greece. Public Health Nutr..

[25] Monteiro C.A., Cannon G., Levy R.B., Moubarac J.-C., Louzada M.L.C., Rauber F., Khandpur N., Cediel G., Neri D., Martinez-Steele E. (2019). Ultra-processed foods: What they are and how to identify them. Public Health Nutr..

[26] Popkin B.M., Barquera S., Corvalan C., Hofman K.J., Monteiro C., Ng S.W., Swart E.C., Taillie L.S. (2021). Towards unified and impactful policies to reduce ultra-processed food consumption and promote healthier eating. Lancet Diabetes Endocrinol..

[27] Elizabeth L., Machado P., Zinöcker M., Baker P., Lawrence M. (2020). Ultra-Processed Foods and Health Outcomes: A Narrative Review. Nutrients.

[28] Forde C.G., Mars M., De Graaf K. (2020). Ultra-Processing or Oral Processing? A Role for Energy Density and Eating Rate in Moderating Energy Intake from Processed Foods. Curr. Dev. Nutr..

[29] Godos J., Bonaccio M., Al-Qahtani W.H., Marx W., Lane M.M., Leggio G.M., Grosso G. (2023). Ultra-Processed Food Consumption and Depressive Symptoms in a Mediterranean Cohort. Nutrients.

[30] Gómez-Donoso C., Sánchez-Villegas A., Martínez-González M.A., Gea A., de Deus Mendonça R., Lahortiga-Ramos F., Bes-Rastrollo M. (2020). Ultra-processed food consumption and the incidence of depression in a mediterranean cohort: The SUN Project. Eur. J. Nutr..

[31] Adjibade M., Julia C., Allès B., Touvier M., Lemogne C., Srour B., Hercberg S., Galan P., Assmann K.E., Kesse-Guyot E. (2019). Prospective association between ultra-processed food consumption and incident depressive symptoms in the French NutriNet-Santé cohort. BMC Med..

[32] Silva C.F.M., Saunders C., Peres W., Folino B., Kamel T., dos Santos M.S., Padilha P. (2021). Effect of ultra-processed foods consumption on glycemic control and gestational weight gain in pregnant with pregestational diabetes mellitus using carbohydrate counting. PeerJ.

[33] Champely S. (2020). pwr: Basic Functions for Power Analysis. R Package, Version 1.3-0. https://CRAN.R-project.org/package=pwr.

[34] World Health Organization Definition, Diagnosis and Classification of Diabetes Mellitus. https://www.staff.ncl.ac.uk/philip.home/who_dmc.htm.

[35] Gilmore L.A., Redman L.M. (2015). Weight gain in pregnancy and application of the 2009 IOM guidelines: Toward a uniform approach. Obesity.

[36] Cox J.L., Holden J.M., Sagovsky R. (1987). Detection of postnatal depression. Development of the 10-item Edinburgh Postnatal Depression Scale. Br. J. Psychiatry.

[37] Leonardou A.A., Zervas Y.M., Papageorgiou C.C., Marks M.N., Tsartsara E.C., Antsaklis A., Christodoulou G.N., Soldatos C.R. (2009). Validation of the Edinburgh Postnatal Depression Scale and prevalence of postnatal depression at two months postpartum in a sample of Greek mothers. J. Reprod. Infant Psychol..

[38] Vivilaki V.G., Dafermos V., Kogevinas M., Bitsios P., Lionis C. (2009). The Edinburgh Postnatal Depression Scale: Translation and validation for a Greek sample. BMC Public Health.

[39] Bountziouka V., Bathrellou E., Giotopoulou A., Katsagoni C.N., Bonou M., Vallianou N., Barbetseas J., Avgerinos P., Panagiotakos D. (2012). Development, repeatability and validity regarding energy and macronutrient intake of a semi-quantitative food frequency questionnaire: Methodological considerations. Nutr. Metab. Cardiovasc. Dis..

[40] Monteiro C.A., Cannon G., Moubarac J.-C., Levy R.B., Louzada M.L.C., Jaime P.C. (2017). The UN Decade of Nutrition, the NOVA food classification and the trouble with ultra-processing. Public Health Nutr..

[41] Papathanasiou G., Georgoudis G., Papandreou M., Spyropoulos P., Georgakopoulos D., Kalfakakou V., Evangelou A. (2009). Reliability measures of the short International Physical Activity Questionnaire (IPAQ) in Greek young adults. Hell. J. Cardiol..

[42] Buysse D.J., Reynolds C.F., Monk T.H., Berman S.R., Kupfer D.J. (1989). The Pittsburgh Sleep Quality Index: A new instrument for psychiatric practice and research. Psychiatry Res..

[43] Kotronoulas G.C., Papadopoulou C.N., Papapetrou A., Patiraki E. (2011). Psychometric evaluation and feasibility of the Greek Pittsburgh Sleep Quality Index (GR-PSQI) in patients with cancer receiving chemotherapy. Support. Care Cancer.

[44] Henshaw E.J. (2023). Breastfeeding and Postpartum Depression: A Review of Relationships and Potential Mechanisms. Curr. Psychiatry Rep..

[45] Dehghan-Banadaki S., Hosseinzadeh M., Madadizadeh F., Mozaffari-Khosravi H. (2023). Empirically derived dietary patterns and postpartum depression symptoms in a large sample of Iranian women. BMC Psychiatry.

[46] Yang C., Zhao A., Lan H., Ren Z., Zhang J., Szeto I.M.-Y., Wang P., Zhang Y. (2021). Association Between Dietary Quality and Postpartum Depression in Lactating Women: A Cross-Sectional Survey in Urban China. Front. Nutr..

[47] Meller F.O., Costa C.d.S., Quadra M.R., Miranda V.I.A., Eugênio F.D., da Silva T.J., Teixeira M.V.R., Schäfer A.A. (2024). Consumption of ultra-processed foods and mental health of pregnant women from the South of Brazil. Br. J. Nutr..

[48] Lassale C., Batty G.D., Baghdadli A., Jacka F., Sánchez-Villegas A., Kivimäki M., Akbaraly T. (2019). Healthy dietary indices and risk of depressive outcomes: A systematic review and meta-analysis of observational studies. Mol. Psychiatry.

[49] Witkowska-Zimny M., Zhyvotovska A., Isakov R., Boiko D., Nieradko-Iwanicka B. (2024). Maternal Sleeping Problems Before and After Childbirth—A Systematic Review. Int. J. Women’s Health.

[50] McEvoy K.M., Rayapati D., Cole K.O.W., Erdly C., Payne J.L., Osborne L.M. (2019). Poor Postpartum Sleep Quality Predicts Subsequent Postpartum Depressive Symptoms in a High-Risk Sample. J. Clin. Sleep Med..

[51] Iranpour S., Kheirabadi G.R., Esmaillzadeh A., Heidari-Beni M., Maracy M.R. (2016). Association between sleep quality and postpartum depression. J. Res. Med. Sci..

[52] Dias C.C., Figueiredo B. (2015). Breastfeeding and depression: A systematic review of the literature. J. Affect. Disord..

[53] Pope C.J., Mazmanian D. (2016). Breastfeeding and Postpartum Depression: An Overview and Methodological Recommendations for Future Research. Depression Res. Treat..

[54] Figueiredo B., Canário C., Field T. (2014). Breastfeeding is negatively affected by prenatal depression and reduces postpartum depression. Psychol. Med..

[55] Hamdan A., Tamim H. (2012). The Relationship between postpartum depression and breastfeeding. Int. J. Psychiatry Med..

[56] Sánchez-Villegas A., Martínez-González M.A., Estruch R., Salas-Salvadó J., Corella D., Covas M.I., Arós F., Romaguera D., Gómez-Gracia E., Lapetra J. (2013). Mediterranean dietary pattern and depression: The PREDIMED randomized trial. BMC Med..

